# A simplified machine learning model utilizing platelet-related genes for predicting poor prognosis in sepsis

**DOI:** 10.3389/fimmu.2023.1286203

**Published:** 2023-11-20

**Authors:** Yingying Diao, Yan Zhao, Xinyao Li, Baoyue Li, Ran Huo, Xiaoxu Han

**Affiliations:** National Clinical Research Center for Laboratory Medicine, Department of Laboratory Medicine, The First Hospital of China Medical University, Shenyang, China

**Keywords:** machine learning, random forest, sepsis, prognosis, platelet-related genes

## Abstract

**Background:**

Thrombocytopenia is a known prognostic factor in sepsis, yet the relationship between platelet-related genes and sepsis outcomes remains elusive. We developed a machine learning (ML) model based on platelet-related genes to predict poor prognosis in sepsis. The model underwent rigorous evaluation on six diverse platforms, ensuring reliable and versatile findings.

**Methods:**

A retrospective analysis of platelet data from 365 sepsis patients confirmed the predictive role of platelet count in prognosis. We employed COX analysis, Least Absolute Shrinkage and Selection Operator (LASSO) and Support Vector Machine (SVM) techniques to identify platelet-related genes from the GSE65682 dataset. Subsequently, these genes were trained and validated on six distinct platforms comprising 719 patients, and compared against the Acute Physiology and Chronic Health Evaluation II (APACHE II) and Sequential Organ-Failure Assessment (SOFA) score.

**Results:**

A PLT count <100×10^9^/L independently increased the risk of death in sepsis patients (OR = 2.523; 95% CI: 1.084-5.872). The ML model, based on five platelet-related genes, demonstrated impressive area under the curve (AUC) values ranging from 0.5 to 0.795 across various validation platforms. On the GPL6947 platform, our ML model outperformed the APACHE II score with an AUC of 0.795 compared to 0.761. Additionally, by incorporating age, the model’s performance was further improved to an AUC of 0.812. On the GPL4133 platform, the initial AUC of the machine learning model based on five platelet-related genes was 0.5. However, after including age, the AUC increased to 0.583. In comparison, the AUC of the APACHE II score was 0.604, and the AUC of the SOFA score was 0.542.

**Conclusion:**

Our findings highlight the broad applicability of this ML model, based on platelet-related genes, in facilitating early treatment decisions for sepsis patients with poor outcomes. Our study paves the way for advancements in personalized medicine and improved patient care.

## Introduction

Sepsis is a serious condition that arises from a systemic inflammatory reaction in the host, leading to immunological dysregulation and potentially life-threatening organ failure (1). In 2017, sepsis infected 48·9 million individuals worldwide, resulting in 11 million deaths, which accounted for 19·7% of all global deaths (2). Severe sepsis is estimated to occur in at least 20·16 million cases, resulting in a minimum of 190,000 deaths (3, 4). In China, sepsis affects one in every five ICU patients, with a 30-day mortality rate of 29·5% and a 90-day mortality rate of 35·5% (5, 6). In contrast, the average 30-day mortality rate in Europe and North America is 24·4% (7). These statistics highlight the higher sepsis mortality rates in China compared to Europe and North America.

Early detection and treatment play a pivotal role in improving the survival rate of patients with poor prognoses. Various scoring systems, such as the Acute Physiology and Chronic Health Evaluation II (APACHE II), Sequential Organ-Failure Assessment (SOFA), and others, have been developed to aid in determining the prognosis of sepsis patients. However, these systems are limited in their clinical usefulness due to their complexity. Some researchers have developed machine learning models for sepsis prognosis using genes (8, 9), but the lack of extensive validation across different microarray platforms hinders the assurance of their reliability and universality. Consequently, there is an urgent and pressing need to develop a simplified and universally applicable prognosis prediction system for clinical applications.

A low platelet count is an independent risk factor for poor outcomes in patients with sepsis (10–12). Thrombocytopenia, defined as a low platelet count, is prevalent in individuals with severe sepsis, occurring in 15-50% of patients with sepsis and septic shock (11, 13, 14). Clausius et al. divided 931 sepsis patients into four groups based on their platelet count at admission and found that those with a platelet count <100×10^9^/L had a greater risk of mortality, and a low platelet count was strongly associated with an increase in 1-year mortality (13). Additionally, the platelet count of non-survivors recovers at a slower rate or not at all compared to survivors after acute sickness (15). These findings suggest a strong association between platelets and sepsis outcomes.

Platelet-related gene loci are involved in regulating platelet production (16), and their mRNA expression in illnesses reflects platelet condition (17). Lydia et al. analyzed the transcriptomes of 180 patients with sepsis whose primary disease was community pneumonia and found that platelet mRNA expression increased in patients with less severe sepsis but decreased as the disease progressed (18). Kim et al. investigated six GEO datasets and found that the platelet pathway was activated in sepsis survivors but not in non-survivors (19). These findings suggest that platelet-related gene expression is associated with sepsis prognosis. Gene expression profiling can provide extensive information on gene expression, and machine learning can be used to extract hidden information from large amounts of data. However, few studies have applied machine learning techniques to investigate whether platelet-related genes can predict sepsis prognosis.

This study aimed to develop a simplified machine learning model based on platelet-related genes to predict sepsis prognosis. For the first time, data from six different microarray platforms were used to validate the model, demonstrating its universality and robustness. Moreover, by comparing the model to the APACHE II and SOFA scores, it was shown to have strong prognostic efficacy in sepsis prognosis. Finally, predicting the molecular interactions between platelet-related genes and antiplatelet drug (aspirin, clopidogrel and indobufen) provided a foundation for future treatment strategies.

## Methods

### Clinical data collection

We conducted a retrospective cohort study and collected clinical information on 365 patients with positive blood cultures from the microbiological database of the First Affiliated Hospital of China Medical University between January 2017 and June 2019. The inclusion criteria were based on documented or suspected infection, the presence of systemic signs and symptoms of inflammation, and positive blood culture results (20). Exclusion criteria were patients under the age of 18 and those with complex bacterial infections. The study was approved by the Ethics Committee of the First Affiliated Hospital of China Medical University. The 365 patients were divided into two groups based on different laboratory data: white blood cell (WBC) count > 9·5×109/L, hemoglobin <100 g/L, platelet count < 100×109/L, C-reactive protein (CRP) > 8 mg/L, and procalcitonin > 0·5μg/L. The elderly group was divided based on age, with those aged ≥60 years considered as elderly.

### Ethics

A retrospective analysis was conducted on data from the First Affiliated Hospital of China Medical University. The Institutional Review Board of the hospital granted an exemption for this study.

### Platelet genetic data collection

The mRNA expression data and associated clinical data for platelet genetics were collected from the GEO and ArrayExpress databases using the search term “sepsis”. The datasets were selected based on the following criteria: (1) research conducted on “Homo sapiens” using array expression profiling, (2) whole blood or white blood cells used as tissue sources, (3) clinical data on death and survival, and (4) at least ten samples included in each dataset. We selected 11 datasets (**Table 1**), with the GSE65682 dataset serving as the training set and the remaining datasets serving as the testing set. The annotation file provided by the microarray manufacturer was used to match each probe to a gene symbol. The Robust Multiple Array (RMA) algorithm was used to background-correct and standardize the microarray raw data (21).

**Table 1:** Filtered public datasets.

Accession	Patients	Country	Timing of mortality	Mortality	Platform
GSE65682	ICU patients with suspected CAP, HAP.	Netherlands	28-day	114/479	GPL13667
GSE48080	Male ICU patient suffering from CAP.	Brazil	NA	5/10	GPL4133
GSE134347	ICU patients	Netherlands	28-day	77/156	GPL17586
GSE95233	ICU patients with septic shock identified according to the diagnostic criteria of the American College of Chest Physicians/Society of Intensive Care Medicine	France	28-day	17/51	GPL570
GSE33118	Septic shock	France	NA	10/20	GPL570
GSE33119	Septic shock due to pneumonia	France	NA	10/20	GPL570
GSE106878	Septic shock	Israel	NA	13/47	GPL10295
GSE54514	ICU patients with sepsis	Australia	NA	9/35	GPL6947
E-MTAB-4451	ICU patients with sepsis caused by CAP	UK	28-day	52/106	GPL10558
E-MTAB-5273	Patients with sepsis caused by CAP and FP	UK	28-day	15/72	GPL10558
E-MTAB-5274	Patients with sepsis caused by CAP and FP	UK	28-day	14/106	GPL10558

CAP, community acquired pneumonia; HAP, hospital acquired pneumonia; FP, fecal peritonitis.

### Platelet signature gene selection

We conducted a stepwise regression analysis on platelet-related genes, using a significance threshold of α=0·05 and discarding non-significant genes at each step (18), to identify genes associated with sepsis-related mortality. The selected genes were further screened using two machine learning techniques, Least Absolute Shrinkage and Selection Operator (LASSO) and Support Vector Machine (SVM), to reduce the dimensionality of the dataset. LASSO uses a parameter reduction method to select features, and we used the glmnet program with 10-fold cross-validation to identify the most relevant genes (22). The random forest algorithm’s recursive feature elimination (RFE) is a supervised machine learning method that iteratively removes the least important features based on model performance until the required features are selected (23).

### Analysis of gene set enrichment

Gene Set Enrichment Analysis (GSEA) is a powerful tool that can rank all genes to predict changes in gene expression between two groups (24). We retrieved canonical pathways containing 3090 gene sets from the MSigDB website at https://www.gsea-msigdb.org/gsea/msigdb/. We used the “limma” package in R to identify differentially expressed genes between the survivor and non-survivor groups, or between high and low expression groups, and then used GSEA to compare the differences in canonical pathways. A significance threshold of P<0·05 and a false discovery rate (FDR) of 25% were used to identify significantly enriched pathways.

### Construction of a model of prognosis for individuals with sepsis based on platelet genes

We used the mlr3 package to evaluate three machine learning models: logistic regression (LR), decision tree (DT), and random forest (RF). LR is a well-established linear prediction technique that has been widely used in recent years for classification problems in medicine (25). DT is a supervised learning algorithm that can effectively handle large amounts of medical data by selecting the most informative attributes to make predictions (26). RF is a popular machine learning method that generates multiple decision trees by randomly selecting features and training samples, and then combines the results of these trees to make final predictions. It is often used for building classification models (27). In this study, we used sepsis-related mortality as the response variable and platelet-related signature genes as the explanatory variables. We randomly divided all samples with clinical outcomes from the GSE65682 dataset into training (70%) and validation (30%) sets for 5-fold cross-validation. We measured the predictive performance of the machine learning models using the area under the receiver operating characteristic (ROC) curve.

### Validated on a wide range of microarray platforms

We obtained external datasets from the GEO (https://www.ncbi.nlm.nih.gov/geo/) and ArrayExpress (https://www.ebi.ac.uk/arrayexpress/) databases. The GEO dataset consisted of platforms GPL4133 (GSE48080), GPL17586 (GSE134347), GPL570 (GSE95233/GSE33118/GSE33119), GPL10295 (GSE106878), and GPL6947 (GSE54514). The ArrayExpress dataset was based on platform GPL10558 (E-MTAB-4451/E-MTAB-5273/E-MTAB-5274). Prior to model verification, datasets from the same platform were normalized using the “sva” R package.

### Molecular docking

To evaluate the binding capacity of antiplatelet drug (aspirin, clopidogrel and indobufen) and associated proteins, molecular docking was performed using AutoDock4 (v 4·2·6). Molecular dynamics simulation was not included in this study, which focused solely on molecular docking. The reliability of docking and accuracy of ligand placement were assessed using the binding energy (BE). The 3D docking visualization was done using PyMOL (v 2·2·0).

### Other statistical analysis

All statistical analyses were performed using R software (version 4·2·3). The Chi-square test, t-test, or Mann-Whitney test were used to examine statistical demographic characteristics and laboratory test results. Univariate and multivariate Cox regression analyses were used to identify independent risk factors that influence the prognosis of sepsis patients. A p-value of less than 0·05 was considered statistically significant.

## Results

### Thrombocytopenia as a risk factor for mortality

Out of the 365 patients with sepsis, 58 (15·9%) died during hospitalization, while 307 were discharged. Of the total patients, 248 were men and 117 were women. **Table 2** shows that among the patients, 2·7% had malignant tumors (excluding blood tumors), 9% had blood diseases (including blood tumors), and 35·3% were surgical patients with sepsis. While it is established that individuals with diabetes face an increased risk of infection, the impact of diabetes on sepsis outcomes and the underlying mechanisms involved remain subjects of ongoing debate (28). This study, however, did not find any evidence of a detrimental effect of diabetes on sepsis outcomes. Nonetheless, our analysis did reveal that hypertension was associated with a higher proportion of non-surviving patients compared to the surviving group.

**Table 2:** 365 patients’ clinical characteristics and laboratory findings.

Variable	All	Group		P value
		Dead	Discharged	
Clinical characteristics				
Age, yrs	61·0 (50·0-69·5)	59·5 (52·5-72·5)	61·0 (50·0-69·0)	0·767
Male	248/365 (67·9)	48/58 (82·8)	200/307 (65·1)	0·008
Prior medical history				
Diabetes	70/365 (19·2)	12/58 (20·7)	58/307 (18·9)	0.436
Hypertension	82/365 (22·5)	19/58 (32·8)	63/307 (20·5)	0.033
Malignancy	15/365 (2·7)	1/58 (1·7)	14/307 (4·6)	0·318
Hematological disease	33/365 (9·0)	10/58 (17·2)	23/307 (7·5)	0·018
Surgical complications	129/365 (35·3)	14/58(24·1)	115/307 (37·5)	0·052
Others	188/365 (51·5)	33/58 (56·9)	155/307 (50·5)	0·371
Laboratory findings				
WBC count, ×10^9^/L	9·6 (5·6-13·9)	8·5 (3·2-14·0)	9·7 (5·9-13·9)	0·285
Hemoglobin, g/L	106 (86-125)	93 (67-115)	108 (89-127)	<0·0001
Platelet count, ×10^9^/L	166·0 (102·0-260·0)	106·0 (34·8-189·3)	176·0 (114·0-270·0)	<0·0001
CRP, mg/L	99·7 (50·9-166·1)	99·7 (58·3-210·6)	97·9 (49·4-162·3)	0·226
PCT,μg/L	1·22 (0·3-14·4)	3·4 (0·8-16·4)	0·9 (0·3-12·4)	0·156

Data are presented as median (IOR) or n/total(%). P value means the comparison between dead group and discharged group of sepsis; WBC, white blood cell; CRP, C-reactive protein; PCT procalcitonin.

Univariate logistic analysis was performed to examine the demographic factors and laboratory test results, as presented in **Table 3**. Male sex (OR = 2·568), lower hemoglobin (OR = 2·577), lower platelets (OR = 3·147), and higher procalcitonin (OR = 2·507) were all found to increase the likelihood of mortality. Further, when these four significant predictors of mortality were included in the multivariate logistic regression model, it was revealed that sepsis patients with PLT<100x10^9^/L had a higher risk of death (OR = 2·523; 95% CI: 1·084-5·872), which is an independent risk factor for death in sepsis patients.

**Table 3:** Analysis of sepsis prognostic factors.

	Number of patients	Proportion (%)	Univariate Cox analysis		Multivariate Cox analysis	
			HR (95% CI for HR)	P value	HR (95% CI for HR)	P value
Sex				0·010		0·104
Female	117	32·1	1·00		1·00	
Male	248	67·9	2·57 (1·25-5·28)		2·26 (0·85-6·01)	
Age				0·178		
≥60	202	55·3	1·00			
<60	163	44·7	0·68 (0·39-1·19)			
WBC				0·817		
>9·5	183	50·1	1·00			
≤9·5	182	49·9	0·94 (0·53-1·64)			
Hemoglobin				0·001		0·366
>90	255	69·9	1·00		1·00	
≤90	110	30·1	2·56 (1·44-4·55)		1·47 (0·64-3·36)	
Platelet				<0·0001		0·032
>100	276	75·6	1·00		1·00	
≤100	89	24·4	3·15 (1·75-5·66)		2·52 (1·08-5·87)	
CRP				0·812		
>8	184	95·3	1·00			
≤8	9	4·7	0·82 (0·16-4·13)			
PCT				0·042		0·147
>0·5	127	63·8	2·51 (1·03-6·09)		1·99 (0·79-5·05)	
≤0·5	72	36·2	1·00		1·00	

### Identification of five platelet-related genes as potential prognostic indicators for sepsis

**Figure 1:**
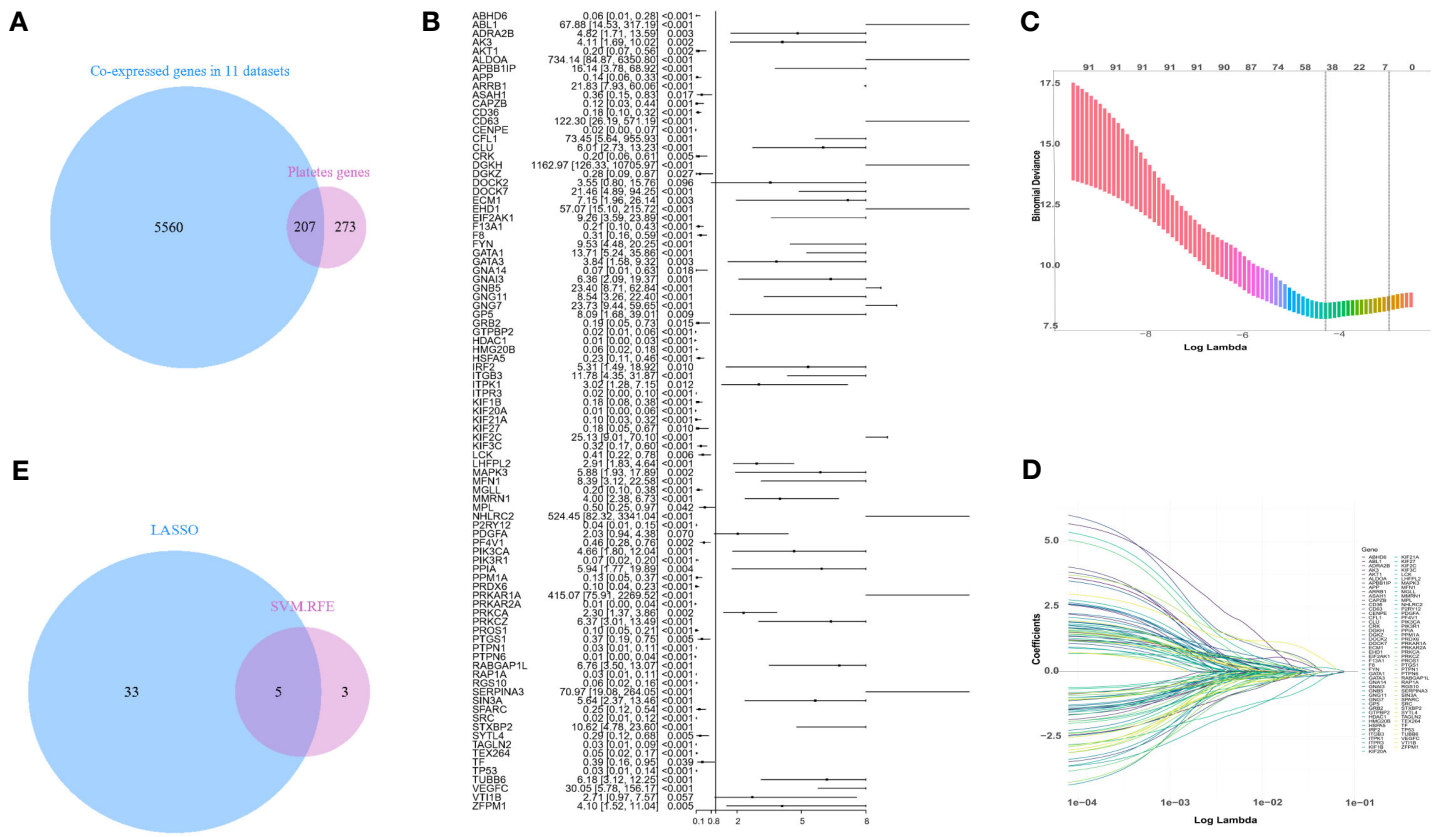
Screening for platelet-related genes linked to sepsis prognosis. **(A)** Platelet-related genes expressed in the training set GSE65682. **(B)** In the training set, a forest plot of platelet-related genes correlated with prognosis. **(C, D)** LASSO-screened feature genes. **(E)** LASSO and SVM-RFE search for platelet-related genes that are shared.

To develop a machine model that is compatible with multiple microarray platforms, we initially merged data from seven microarray platforms, comprising a total of eleven datasets. This consolidation allowed us to acquire 5767 gene expression values that were collectively expressed across seven microarray platforms. 480 platelet-related genes were collected from the GSEA (29). Through Venn analysis of the 5767 co-expressed genes and the 480 platelet-related genes, we identified a total of 207 genes that were both platelet-related and co-expressed (**Figure 1A**).

In the training set GSE65682, a Cox model was fitted to the initial set of 207 genes, gradually eliminating less significant genes. Eventually, 91 genes were strongly correlated with mortality, as depicted in **Figure 1B**. To further reduce the dimensionality of the data, LASSO and SVM-RFE algorithms were employed to identify additional crucial genes associated with mortality. LASSO employed 10-fold cross-validation to adjust penalized parameters and selected 38 distinct genes from the pool of 91 genes (**Figures 1C, D**). Meanwhile, SVM-RF identified eight genes. By performing a Venn analysis on the co-expressed genes identified by both approaches (**Figure 1E**), a total of five important genes were identified: *GTPBP2*, *ALDOA*, *PRKAR2A*, *KIF2C*, and *NHLRC2*.

### Implication of five genes in platelet signaling pathway regulation

**Figure 2:**
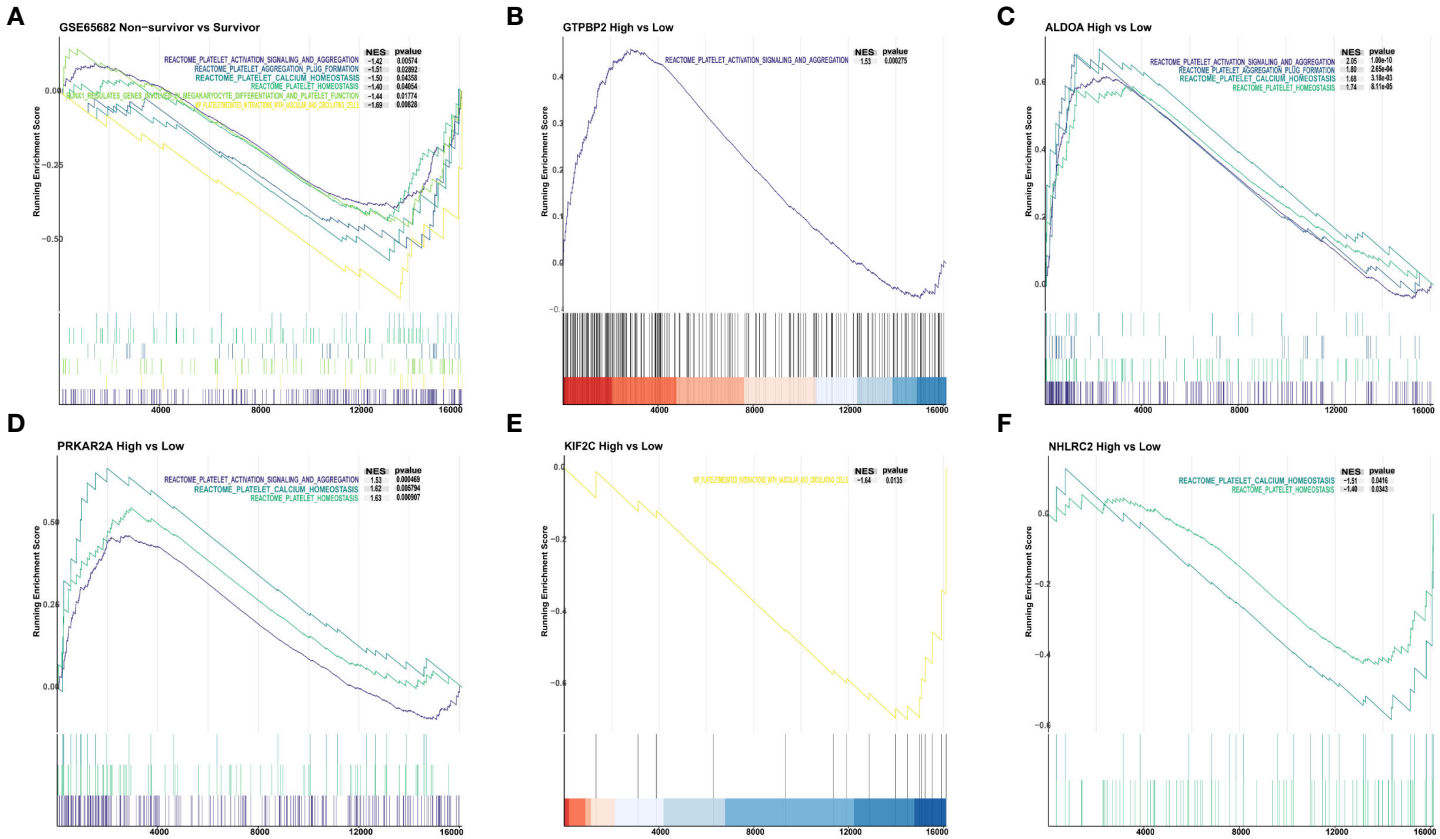
The connection between 5 genes and the platelet classical pathway. **(A)** Platelet pathways differ between non-survivors and survivors. **(B)** Platelet pathway differences between *GTPBP2* high and low expression groups. **(C)** Platelet pathway differences between the *ALDOA* high expression and low expression groups. **(D)** Differences in platelet pathways between the *PRKAR2A* high and low expression groups. **(E)** Platelet pathway differences between *KIF2C* high and low expression groups. **(F)** Platelet pathway differences between *NHLRC2* high and low expression groups.

By conducting GSEA on samples with clinical outcomes from the GSE65682 dataset, we identified a total of six platelet signal pathways that were enriched in the survival group. These pathways encompassed various aspects of platelet biology, including platelet activation, signal transduction, and aggregation pathways, as well as platelet-mediated interactions with blood vessels and circulating cells. Additionally, we found that the platelet aggregation (thrombosis) pathway, RUNX1-regulating genes involved in megakaryocyte differentiation, platelet function pathway, platelet calcium homeostasis pathway, and platelet homeostasis pathway were also enriched (**Figure 2A**).

To investigate whether the five identified genes are involved in the regulation of aforementioned pathways, we divided all sepsis samples with clinical outcomes into high and low expression groups based on the median expression levels of the five genes: *GTPBP2*, *ALDOA*, *PRKAR2A*, *KIF2C*, and *NHLRC2*. The *GTPBP2* overexpression group exhibited enrichment in the platelet activation, signal transduction, and aggregation pathways (**Figure 2B**). In the *ALDOA* high expression group, we found enrichment in four pathways: platelet activation, signal transduction, and aggregation pathways, platelet aggregation (thrombosis) pathway, platelet calcium homeostasis pathway, and platelet homeostasis pathway (**Figure 2C**). Similarly, the *PRKAR2A* high expression group showed enrichment in three pathways: platelet activation, signal transduction, and aggregation pathways, platelet calcium homeostasis pathway, and platelet homeostasis pathway (**Figure 2D**). On the other hand, the *KIF2C* low expression group exhibited enrichment specifically in the platelet-mediated interactions with blood vessels and circulating cells pathway (**Figure 2E**). And the *NHLRC2* low expression group displayed enrichment in the platelet calcium homeostasis pathway and platelet homeostasis pathway (**Figure 2F**).

In addition to the pathways mentioned above for the survivor and non-survivor groups, the *GTPBP2* high expression group showed enrichment in the response to elevated platelet cytosolic Ca2+ pathway. The *ALDOA* high expression group exhibited enrichment in the response to elevated platelet cytosolic Ca2+ and platelet sensitization by low-density lipoprotein (LDL) pathways. Similarly, the *PRKAR2A* high expression group showed enrichment in the response to elevated platelet cytosolic Ca2+ and platelet sensitization by LDL pathways (**Figure S1**).

### Development and cross-platform validation of machine learning models for five platelet-related genes

**Figure 3:**
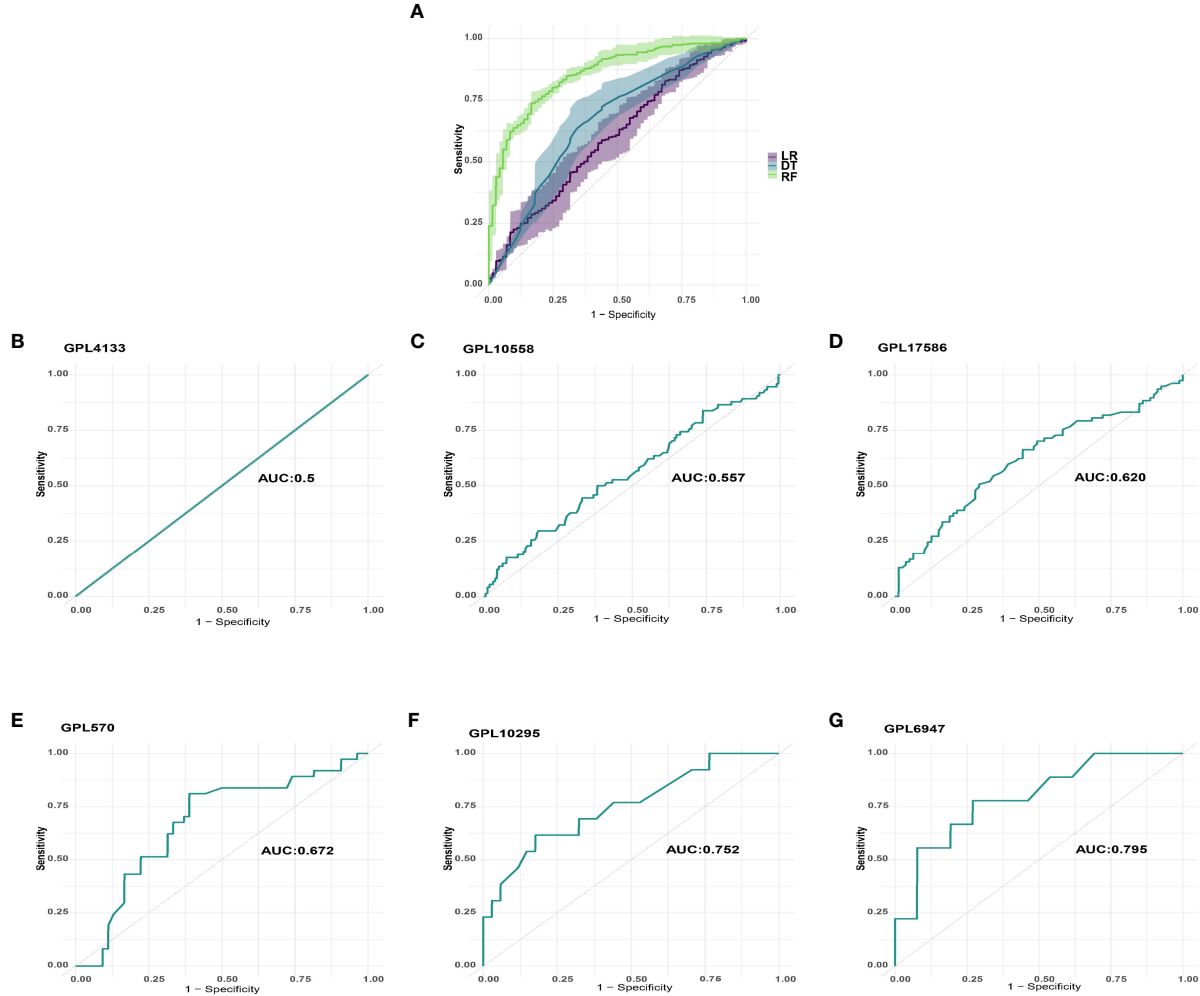
Machine learning model selection and verification. **(A)** LR, DT, and RF models’ training effects in the training set. **(B)** GPL4133 platform’s AUC. **(C)** GPL10558 platform’s AUC. **(D)** GPL17586 platform’s AUC. **(E)** GPL570 platform’s AUC. **(F)** GPL10295 platform’s AUC. **(G)** GPL6947 platform’s AUC.

We utilized GSE65682 as the training set and used the normalized mRNA expression of the five genes as the input variable and the death of sepsis patients as the outcome event to build a prognosis model. **Figure 3** shows the area under the curve (AUC) obtained when using 5-fold cross-validation to compare the training effects of LR (AUC=0·600), DT (AUC=0·664), and RF (AUC=0·858) models in the training set. Finally, RF was chosen to perform prognostic classification on the data.

We examined the predicted effect of the machine learning model across platforms by using prognostic classification on data from the corresponding platforms. To minimize analytical errors, datasets from the same platform were first removed from batch effects. The datasets from six platforms were then standardized and processed for validation using Z-Score. The AUCs of GPL4133 (GSE48080), GPL10558 (E-MTAB-4451/E-MTAB-5273/E-MTAB-5274), GPL17586 (GSE134347), GPL570 (GSE95233/GSE33118/GSE33119), GPL10295 (GSE106878), and GPL6947 (GSE54514) were 0·5, 0·557, 0·620, 0·672, 0·752, and 0·795, respectively (**Figure 3**).

A study has provided evidence that randomly selected genes from the human genome occasionally demonstrate superior prognosis prediction abilities compared to selected gene features (30). To evaluate the performance of the model we constructed, a validation process was conducted. We randomly selected 500 genes and organized them into 100 genomes, with each genome comprising 5 genes. These randomly generated genomes were then employed to develop 100 new machine models, utilizing GSE65682 as the training set. Subsequently, these models were validated on six distinct platforms, allowing us to assess their effectiveness and robustness.

By comparing the area under the curve (AUC) between the model constructed with non-random platelet-related genes and the model constructed with random genes, we observed that, on the majority of platforms, the non-random platelet-related gene model exhibited superior performance compared to 95% of the random gene model, as indicated by a higher area under the curve (AUC). However, it is worth noting that the GPL4133 platform showed different results, as illustrated in **Figure S2**.

### Comparison of sepsis prognosis accuracy between the machine learning model and the APACHE II or SOFA score

To assess the predictive efficacy of the five platelet-related gene models in sepsis patient prognosis, we conducted a comparative analysis with the APACHE II score on the GSE54514 dataset (GPL6947), the APACHE II and the SOFA scores on the GSE48080 dataset (GPL4133). This evaluation aimed to determine the relative performance and effectiveness of the model in predicting outcomes in sepsis patients.

**Figure 4:**
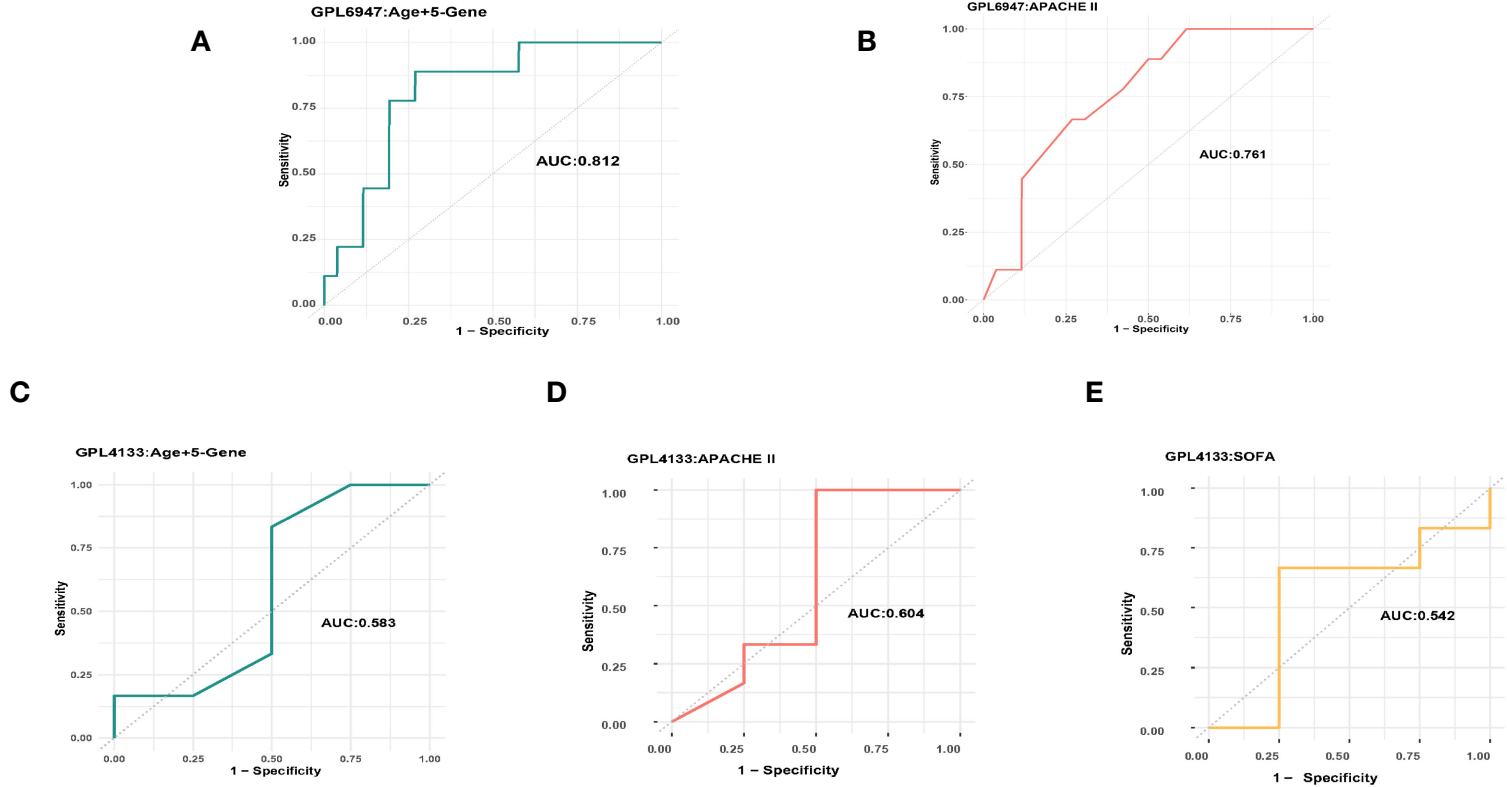
The AUC of the machine learning model and the APACHE II score. **(A)** GPL6947 Age+5-Gene AUC. **(B)** GPL6947 APACHE II AUC. **(C)** GPL4133 Age+5-Gene AUC. **(D)** GPL4133 APACHE II AUC. **(E)** GPL4133 SOFA AUC.

On the GPL6947 platform, the machine learning model based on five platelet-related genes achieved an AUC of 0.795 (**Figure 3G**). By incorporating age into the model, further training resulted in an improved AUC of 0.812 (**Figure 4A**). In comparison, the AUC of the APACHE II score was 0.761 (**Figure 4B**). Notably, on this platform, the machine learning models demonstrated significantly superior performance in predicting the prognosis of sepsis compared to the APACHE II score.

On the GPL4133 platform, the machine learning model based on five platelet-related genes achieved an AUC of 0.5 (**Figure 3B**). However, by incorporating age into the model, the AUC improved to 0.583 (**Figure 4C**). In comparison, the AUC of the APACHE II score was 0.604 (**Figure 4D**), and the AUC of the SOFA score was 0.542 (**Figure 4E**). Remarkably, on the GPL4133 platform, the predictive performance of the machine learning model developed for five platelet-related genes and age aligns closely with the performance of the APACHE II score and SOFA score.

### Platelet related protein and antiplatelet drug interaction

According to the findings, the major genes influencing sepsis prognosis were *GTPBP2*, *ALDOA*, *PRKAR2A*, *KIF2C*, and *NHLRC2*. Since antiplatelet drug (aspirin, clopidogrel and indobufen) possesses antiplatelet and antiaggregation properties, we performed molecular docking of these genes with aspirin using Autodock to confirm their interaction. The 2D structures of antiplatelet drug (aspirin, clopidogrel and indobufen) were available for download on PubChem, while the 3D structures of ALDOA (PDB: 6XML), PRKAR2A (PDB: 5H78), NHLRC2 (PDB: 6GC1), and KIF2C (PDB: 2HEH) were downloaded from the PDB website. As PDB does not provide a 3D structure for GTPBP2, we downloaded the confirmed alpha-fold structure (UNIprotKB identifier: AF-Q9BX10-F1).

**Figure 5:**
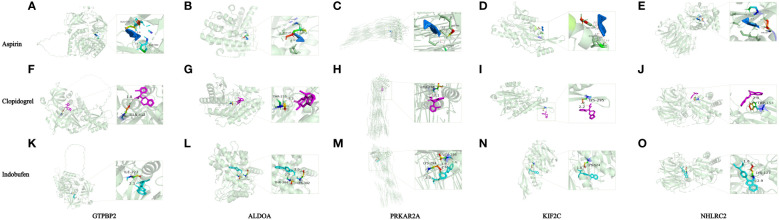
Simulations of protein-ligand interactions for molecular docking. **(A)** Aspirin - GTPBP2. **(B)** Aspirin - ALDOA. **(C)** Aspirin - PRKAR2A. **(D)** Aspirin - KIF2C. **(E)** Aspirin - NHLRC2. **(F)** Clopidogrel -GTPBP2. **(G)** Clopidogrel - ALDOA. **(H)** Clopidogrel - PRKAR2A. **(I)** Clopidogrel - KIF2C. **(J)** Clopidogrel - NHLRC2. **(K)** Indobufen-GTPBP2. **(L)** Indobufen- ALDOA. **(M)** Indobufen - PRKAR2A. **(N)** Indobufen - KIF2C. **(O)** Indobufen - NHLRC2.

Aspirin had binding energies of -3·91, -4·13, -4·32, -3·1, and -3·92 kcal/mol to GTPBP2, ALDOA, PRKAR2A, KIF2C, and NHLRC2, respectively. Clopidogrel (plavix) had binding energies of -3·78, -1·75, -5·43, -0·74, and -4·12 kcal/mol to GTPBP2, ALDOA, PRKAR2A, KIF2C, and NHLRC2, respectively. Indobufen had binding energies of -3·29 -5·47, -4·41, -4·56, and -4·15 kcal/mol to GTPBP2, ALDOA, PRKAR2A, KIF2C, and NHLRC2, respectively. **Figure 5** depicts additional information, such as atomic distances and binding site data. From the above results, Apart from ALDOA and KIF2C, which exhibit slightly weaker binding ability to Clopidogrel, the remaining antiplatelet drugs demonstrate stable binding to these proteins.

## Discussion

The timely diagnosis of sepsis patients with poor prognosis is critical to improve clinical outcomes. In our study, we constructed a machine learning model using five genes, including *GTPBP2*, *ALDOA*, *PRKAR2A*, *KIF2C*, and *NHLRC2*, to identify sepsis patients with a poor prognosis. To the best of our knowledge, this is the first machine learning model to demonstrate its applicability in predicting sepsis prognosis across various microarray platforms, thereby filling a critical research gap. And, the results showed that this model performed more reliably in predicting prognosis compared to the APACHE II or SOFA score. Additionally, molecular docking confirmed that aspirin can stably bind to the proteins associated with these genes, providing a foundation for future treatment.

Since the introduction of the Sepsis-3 standard (1), the predictive importance of platelet count in the prognosis of sepsis patients has been increasingly recognized. Jiang et al. investigated the survival of 120 sepsis patients with urinary tract infections and found that a continuous decrease in platelet count was associated with a poor prognosis in urosepsis patients (31). Similarly, Sinha et al. discovered that platelet count on the first day of admission could predict 28-day mortality in sepsis patients (32). However, these studies were limited to specific sepsis infections or excluded conditions such as hematological disorders and cancer, which are common complications of cancer treatment (33). Excluding cancer patients may introduce bias in the research. To address this issue, Schupp et al. investigated the relationship between platelet count and prognosis in 358 sepsis patients, including those with cancer, and found that a continuous decrease in platelet count was associated with increased 30-day mortality in sepsis and septic shock patients (34). However, this study excluded patients who acquired sepsis following surgery, which accounts for around 30% of sepsis cases (35). Our study focused on a cohort of sepsis patients hospitalized with hematological diseases, cancers other than hematological cancers, postoperative complications, and other diseases. We found that a platelet count <100 x 10^9^/L was an independent risk factor for death in sepsis patients, which is consistent with previous studies.

Although recent studies have highlighted the crucial role of platelet count in determining sepsis prognosis, there is a scarcity of research on the relationship between platelet-related genes and mortality. In our investigation, we identified *GTPBP2*, *ALDOA*, *PRKAR2A*, *NHLRC2*, and *KIF2C* as genes related to sepsis death using three distinct techniques. *GTPBP2* is involved in signal transduction via small GTPases and influences platelet activation (36). Our study revealed that sepsis patients with high *GTPBP2* expression had increased platelet activation, aggregation, and thrombosis, as well as higher levels of intracytoplasmic calcium ions in platelets. *ALDOA* is a platelet activation and degranulation aldolase isoenzyme (37). In our study, *ALDOA* was implicated in platelet activation, aggregation, and thrombus formation, as well as other platelet activation activities. *PRKAR2A* encodes protein kinase A (PKA), and PKA inhibition can cause platelet death and acute platelet depletion, whereas PKA activation can protect platelets from apoptosis and allow them to be removed (38). Our study showed that the *PRKAR2A* high expression group was enriched in platelet activation, aggregation, and thrombus formation, as well as platelet and calcium ion expression in platelets, indicating that *PRKAR2A* is involved in the platelet activation process. *KIF2C* is involved in the development of megakaryocytes and the generation of platelets (39), while the connection between *NHLRC2* and platelets is unknown. Our study revealed that *KIF2C* is also involved in platelet-mediated interactions with blood vessels or circulating cells in sepsis patients, and *NHLRC2* is involved in platelet homeostasis and calcium homeostasis in platelet cytoplasm, although the specific mechanism of these two genes remains unknown.

These five genes are involved in platelet activation, thrombus formation, and platelet interaction with endothelial cells and immune cells. Platelet activation reduces platelet survival, which contributes to the decline in platelet count (40). Therefore, we hypothesize that these five genes influence platelet number by modulating platelet activation and other processes. It has been shown that nonsurvivors of sepsis have various immunosuppressive innate and adaptive immune systems (41). Our study showed that platelet activation, aggregation, and thrombus formation in non-survivors, as well as platelet interaction with circulating endothelial and immune cells, regulation of megakaryocytes by RUNX1 and its partner CBFB, platelet homeostasis, and cytoplasmic calcium homeostasis were all inhibited, consistent with previous research. These findings suggest that these five genes may influence the immunological state and prognosis of sepsis patients by regulating platelet-related pathways. However, further research is necessary to fully understand the mechanisms involved.

Several studies have utilized genomic expression profiles to develop diagnostic and prognostic models for sepsis (42–45). However, as these investigations involve tens or hundreds of genes, they are challenging to apply in clinical practice. Some researchers have developed sepsis prognostic models incorporating only a few genes (8, 9), but the application of various microarray technologies has not been established. Different microarray platforms utilize different materials and methodologies, which may lead to contradictory results and reduce the generalizability of machine learning models.

In this study, we developed a machine learning model based on platelet-related genes and validated it using data from six different platforms. To our knowledge, this is the first time that a machine learning model has been constructed and validated using platelet-related genes across multiple microarray platforms. To further validate the prognostic predictive capability of our model constructed using screened genes, we established a machine model based on random genes. Across all validation platforms, the AUC of most machine models utilizing random genes remained below 0.6. However, machine models based on platelet-related genes, with the exception of the GPL4133 and GPL10558 platforms, achieved an AUC above 0.6 for all validation platforms. Notably, a recent study revealed that even widely used and authoritative critical illness evaluation systems such as APACHE II and SOFA in clinical practice failed to attain an AUC of 0.7 for predicting mortality rates at various time points in sepsis (46). Consequently, the machine model established in this study demonstrates commendable prognostic prediction performance. The low verification effect observed in the GPL4133 platform may be attributed to the small sample size, consisting of only 5 survivors and 5 non-survivors. GPL10558 platform combines multiple standardized datasets. Although these datasets are tested on the same platform, batch effects can still occur, which may introduce bias in the merged data (47). Additionally, the use of different experimental instruments and reagents based on physical and chemical principles, due to different platforms in the training set, can result in poor compatibility of the obtained expression spectra (48). Consequently, the data testing on this platform may yield lower AUC. To address these challenges, recent studies have proposed methods such as quantile normalization and cross platform normalization to mitigate cross-platform bias and batch effects (49) Future studies can employ these methods to further validate the conclusions of this study. Furthermore, we compared AUC of this model with the APACHE II and SOFA scores to evaluate its prognostic predictive effect. A high APACHE II or SOFA score indicates a worsening illness, a poor prognosis, and an increased risk of death (50, 51). In this study, we made an intriguing discovery regarding the prognostic performance of the machine learning model based on five platelet-related genes. Whether on the GPL6947 platform with the highest prediction effectiveness or the GPL4133 platform with the lowest prediction effectiveness, our model consistently demonstrated robust prognostic performance when age was incorporated. Remarkably, the model’s performance was not only comparable to the conventional APACHE II score or SOFA score but even surpassed them in certain cases. Moreover, our model offers the distinct advantage of being more efficient and convenient for implementation in clinical practice.

Lastly, we used molecular docking to assess the interaction capabilities of five major target proteins (GTPBP2, ALDOA, PRKAR2A, NHLRC2, KIF2C) and antiplatelet drug (aspirin, clopidogrel and indobufen). The binding energy range for these five proteins with aspirin, clopidogrel, and indobufen are as follows: -4.32 to -3.1 kcal/mol, -5.43 to -0.74 kcal/mol, and -5.47 to -3.29 kcal/mol, respectively. With the exception of ALDOA and KIF2C, which exhibit slightly weaker binding to Clopidogrel, all other platelet-related target proteins demonstrate stable docking ability with antiplatelet drugs.

Our study has several limitations that must be acknowledged. Firstly, merging the datasets resulted in the exclusion of many genes, potentially resulting in the loss of some significant genes. Further research is required to confirm these findings across multiple microarray platforms. Secondly, to address the low validation effects observed on certain platforms, it is necessary to expand the sample size or validate the machine models using the same batch of results. This will help ensure the reliability and accuracy of the findings. Finally, while data mining tools were utilized to confirm our findings, they must be validated through clinical studies or animal tests.

In conclusion, this study is the first to investigate the prognostic effect of platelet-related genes on sepsis prognosis and validate it across six microarray platforms, comprising a total of 10 datasets. Our research findings demonstrate that our model exhibits prognostic performance that is at least comparable to the classic APACHE II or SOFA scores. However, our model offers the advantage of being more efficient and convenient for application in clinical practice. Additionally, molecular docking studies confirmed that antiplatelet drug can effectively bind to the proteins associated with these genes, providing a promising foundation for future treatment strategies.

## Data availability statement

The datasets presented in this study can be found in online repositories. The names of the repository/repositories and accession number(s) can be found below: Gene Expression Omnibus(GSE65682/GSE48080/GSE134347/GSE95233/GSE33118/GSE33119/GSE106878/GSE54514); ArrayExpress(E-MTAB-4451/E-MTAB-5273/E-MTAB-5274).

## Ethics statement

The studies involving humans were approved by The Institutional Review Board of the First Affiliated Hospital of China Medical University. The studies were conducted in accordance with the local legislation and institutional requirements. The ethics committee/institutional review board waived the requirement of written informed consent for participation from the participants or the participants’ legal guardians/next of kin because The Ethics Committee has decided that retrospective studies do not require informed consent form. Written informed consent was not obtained from the individual(s) for the publication of any potentially identifiable images or data included in this article because The Ethics Committee has decided that retrospective studies do not require informed consent form.

## Author contributions

YD: Writing – original draft, Writing – review and editing. YZ: Data curation, Writing – review and editing. XL: Software, Writing – review and editing. BL: Data curation, Software, Writing – review and editing. RH: Data curation, Software, Writing – review and editing. XH: Writing – review and editing.

## Supplementary material

Supplementary Figure 1 The connection between 3 genes and the platelet classical pathway. **(A)** Platelet pathway differences between *GTPBP2* high and low expression groups. **(B)** Platelet pathway differences between the *ALDOA* high expression and low expression groups. **(C)** Differences in platelet pathways between the *PRKAR2A* high and low expression groups.

Supplementary Figure 2 The distribution of AUC values for both the non-random gene models and the random gene models across six validation platforms. **(A)** GPL4133 platform. **(B)** GPL10558 platform. **(C)** GPL17586 platform. **(D)** GPL570 platform. **(E)** GPL10295 platform. **(F)** GPL6947 platform.
