# Supplementary figures and images for: A simplified machine learning model utilizing platelet-related genes for predicting poor prognosis in sepsis
Source: Front Immunol. 2023 Nov 20;14:1286203. doi: 10.3389/fimmu.2023.1286203 (PMC10694245; doi:10.3389/fimmu.2023.1286203)

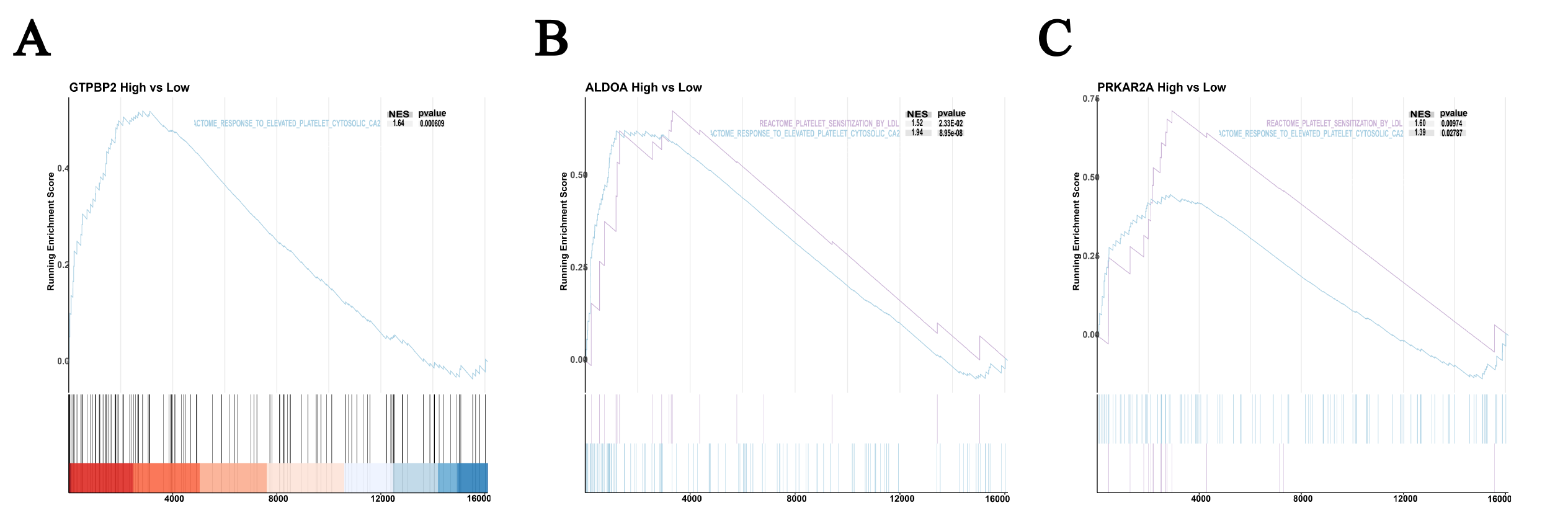

Supplement: Supplementary Figure 1 — The connection between 3 genes and the platelet classical pathway. (A) Platelet pathway differences between GTPBP2 high and low expression groups. (B) Platelet pathway differences between the ALDOA high expression and low expression groups. (C) Differences in platelet pathways between the PRKAR2A high and low expression groups. [file Image_1.tif]

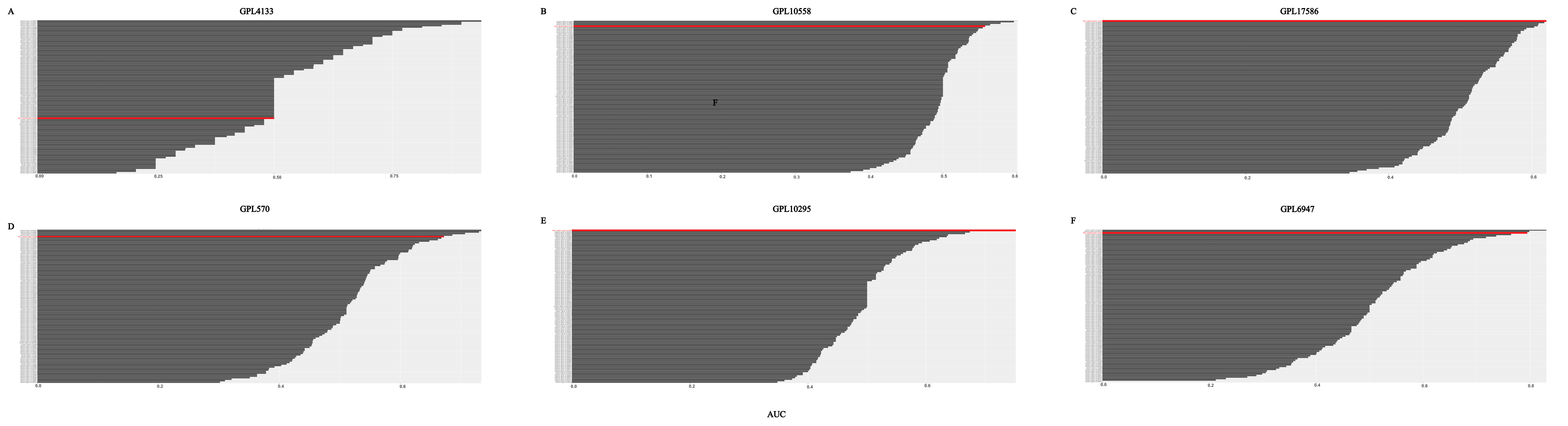

Supplement: Supplementary Figure 2 — The distribution of AUC values for both the non-random gene models and the random gene models across six validation platforms. (A) GPL4133 platform. (B) GPL10558 platform. (C) GPL17586 platform. (D) GPL570 platform. (E) GPL10295 platform. (F) GPL6947 platform. [file Image_2.tif]
